# Malaria prevalence and molecular markers of Plasmodium falciparum antimalarial drug resistance among mobile populations in malaria-endemic countries: A systematic review and meta-analysis

**DOI:** 10.21203/rs.3.rs-9977950/v1

**Published:** 2026-06-28

**Authors:** Alex Mwesigwa, Dianah Fatima, Bienvenu Nsengimaana, Emmanuel Arineitwe, Alison A. Kinengyere, Arthur Mpimbaza, Pauline Byakika-Kibwika, Sam M. Mbulaiteye, Philip J. Rosenthal, Samuel L. Nsobya

**Affiliations:** Kabale University; Mulago Hospital; Infectious Diseases Research Collaboration (IDRC); Infectious Diseases Research Collaboration (IDRC); Makerere University; Makerere University College of Health Sciences; Mbarara University of Science and Technology; National Cancer Institute, National Institutes of Health; University of California; Infectious Diseases Research Collaboration (IDRC)

**Keywords:** Malaria, Plasmodium falciparum, Drug resistance, Mobile populations

## Abstract

**Background:**

Mobile populations in malaria-endemic regions are at increased risk of *Plasmodium falciparum* infection and may contribute to the spread of antimalarial drug resistance across borders. However, evidence on infection prevalence, molecular resistance markers, and associated risk factors in these populations remains limited. This systematic review and meta-analysis synthesized available evidence to inform mobility-responsive malaria surveillance and control strategies.

**Methods:**

A systematic review and meta-analysis were conducted according to PRISMA 2020 guidelines. Studies published between January 2000 and October 2025 reporting *P. falciparum* infection and resistance-associated mutations in PfK13, PfCRT, PfMDR1, PfDHFR, and PfDHPS among mobile populations were included. PubMed, EMBASE, Global Health, and Scopus were searched. Two reviewers independently screened studies, extracted data, and assessed risk of bias. Pooled prevalence estimates were generated using random-effects models in Stata 17, with subgroup analyses by WHO region and population type. Heterogeneity was assessed using I^2^ statistics.

**Results:**

Twenty-six studies involving 7,217 participants from 10 countries were included. Populations studied comprised refugees, migrant workers, travellers, forest-goers, gold miners, and nomadic groups. The pooled prevalence of *P. falciparum* infection was 18% (95% CI: 15–21%), with higher prevalence in Africa (36%) and lower prevalence in South-East Asia (6%). Refugees had the highest pooled prevalence (38%), whereas short-term travellers had lower prevalence (8%). Resistance markers were widely reported, although prevalence varied across settings and time periods. PfK13 mutations were generally infrequent but heterogeneous; validated mutations such as C580Y were repeatedly detected in Myanmar and neighbouring areas of Cambodia and Vietnam, indicating artemisinin resistance hotspots. PfCRT K76T remained common in older studies. PfMDR1 mutations, especially N86Y and Y184F, were frequently reported and reached up to 68% in African refugee populations, although declining trends were noted in recent African studies. PfDHFR and PfDHPS mutations were widely distributed. Infection risk was associated with mobility patterns, occupational exposure, and parasite origin. Substantial heterogeneity was observed (I^2^ = 97.8%).

**Conclusion:**

Mobile populations bear a substantial burden of *P. falciparum* infection and frequently harbor antimalarial drug-resistance markers. Integrating these populations into genomic and cross-border surveillance systems may improve early detection of resistance and strengthen malaria elimination efforts.

## Background

Malaria remains a major global health challenge, with an estimated 282 million cases and 610,000 deaths worldwide in 2024 [1]. *Plasmodium falciparum*, the most virulent human malaria parasite, accounts for approximately 95% of malaria-related morbidity and mortality, particularly in sub-Saharan Africa, but is also present in South-East Asia, Oceania, the Eastern Mediterranean, and Latin America [1, 2]. This persistent burden highlights the need to better understand transmission dynamics alongside emerging threats such as drug resistance and human mobility.

Over the past two decades, malaria control has improved substantially through the scale-up of long-lasting insecticidal nets, indoor residual spraying, and artemisinin-based combination therapies (ACTs) [3–5]. However, progress has recently stalled, in part due to the emergence and spread of antimalarial drug resistance [6]. Artemisinin partial resistance, first reported in South-East Asia [7], has now been identified in several African countries, including Rwanda, Uganda, Tanzania, Ethiopia, and Eritrea [8–13]. This resistance is primarily associated with mutations in the propeller domain of the PfK13 gene[14]. While its clinical implications in high-transmission African settings remain incompletely understood, these evolving resistance patterns may have profound public health relevance beyond acute malaria outcomes. Crucially, by extending the duration of parasitemia, resistance can exacerbate chronic *P. falciparum* exposure, a primary driver, alongside Epstein–Barr virus co-infection, in the pathogenesis of Burkitt lymphoma (BL) [15].

Resistance to some ACT partner drugs is mediated by molecular alterations in key parasite proteins, including PfCRT, which mediates drug efflux from the food vacuole, and PfMDR1, which modulates transport of drugs into the food vacuole. Amplification of *pfpm2* and *pfpm3* genes and novel mutations in PfCRT is associated with piperaquine resistance, while mutations in PfDHFR and PfDHPS confer resistance to sulfadoxine–pyrimethamine [16]. The co-occurrence of multiple mutations may result in multidrug resistance, posing a significant threat to current treatment strategies and highlighting the need for robust molecular surveillance.

Human mobility is an important driver of malaria transmission and the spread of drug-resistant parasites [17–20]. Mobile populations, defined as individuals who move temporarily, seasonally, or permanently for work, livelihood, or displacement include migrant workers, nomadic pastoralists, refugees, internally displaced persons, traders, and forest workers [18]. These groups often have limited access to healthcare and are underrepresented in routine surveillance systems. They may carry asymptomatic or submicroscopic infections and move between areas with differing transmission intensities and treatment practices, facilitating the introduction and spread of resistant parasites [21–26]. For example, studies in Uganda have shown that *P. falciparum* infections among refugees arriving from South Sudan and the Democratic Republic of Congo carry drug resistance markers at prevalences different from those in local populations, enabling importation of resistant strains [27].

Despite growing recognition of the potential role of human mobility in malaria transmission and resistance dynamics [19], comprehensive data on *P. falciparum* infection among mobile populations remain limited. Most reviews have focused on international travellers and refugees [28, 29], with little attention to the other mobile groups. Furthermore, there is limited synthesis integrating molecular resistance data with epidemiological risk factors across diverse mobile populations. To address these gaps, we conducted a systematic review and meta-analysis to synthesize evidence on the prevalence of *P. falciparum* infection and molecular markers of drug resistance among mobile populations in malaria-endemic regions. We focused on amino acid substitutions mediating antimalarial resistance in PfK13, PfCRT, PfMDR1, PfDHFR, and PfDHPS [30, 31]. By integrating epidemiological and molecular data, we aimed to provide a more comprehensive understanding of the role of mobility in malaria transmission and resistance spread.

## Methods

### Study design and protocol registration

This systematic review and meta-analysis were conducted in accordance with the Preferred Reporting Items for Systematic Reviews and Meta-Analyses (PRISMA) 2020 guidelines [32]. The review protocol was registered in the International Prospective Register of Systematic Reviews (PROSPERO) under registration number CRD420251122226. The review question and eligibility criteria were developed using the PECOST (Population, Exposure, Comparator, Outcomes, Settings, and Time) framework, as summarized in Table 1.

### Eligibility criteria

Studies published in English between January 2000 and October 2025 were included if they reported the primary outcome and at least one secondary outcome among mobile populations. Eligible studies included cross-sectional studies, cohort studies, surveillance reports, and randomized controlled trials. Studies were excluded if outcomes were not disaggregated for mobile populations or if they were reviews, editorials, case reports, or conference abstracts without primary data. Mobile populations were categorized as defined in Table 2, and these definitions informed data extraction, subgroup analyses, and interpretation of prevalence and resistance patterns.

### Search strategy and information sources

A systematic literature search was conducted in accordance with PRISMA 2020 guidelines [33]. An experienced medical librarian (AAK) developed and executed the search strategy in collaboration with the review team. The following electronic databases were searched: PubMed, EMBASE, Global Health, and Scopus. Additionally, the reference lists of included studies were screened to identify additional eligible records. The search strategy incorporated a combination of Medical Subject Headings (MeSH) and free-text keywords related to *Plasmodium falciparum*, antimalarial drug resistance proteins (PfK13, PfCRT, PfMDR1, PfDHFR, PfDHPS), mobile populations, migration, cross-border movement, diagnostic methods, and malaria prevalence. Boolean operators “AND” and “OR” were used to structure the search queries [34]. Synonyms and related terms for each major concept were combined using “OR” to ensure comprehensive retrieval of relevant literature, while distinct concepts were combined using “AND” to refine the results to studies addressing all key elements of interest. Parentheses were applied to ensure proper nesting and logical coherence across database platforms. All references retrieved were imported into EndNote X9 for deduplication. The complete search strategies for each database are provided in additional file 1, Table S1.

### Article screening

**Figure 1:**
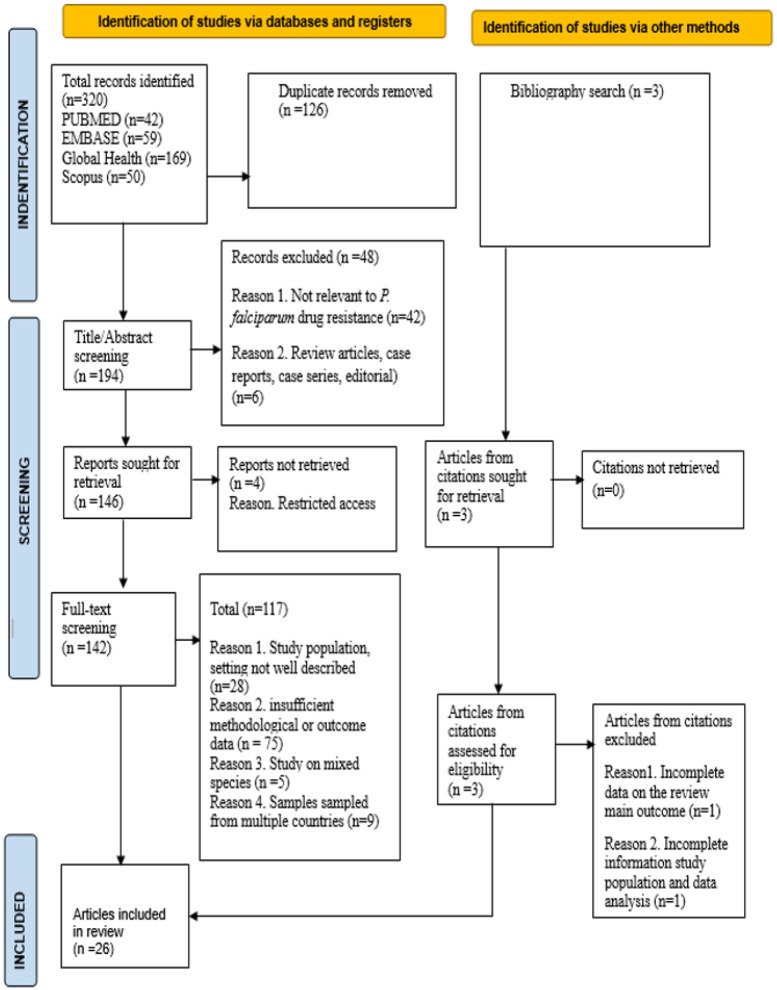
PRISMA Flow diagram for identification of articles included in the review.

Two independent reviewers (AM and BN) screened all titles and abstracts for relevance according to predefined eligibility criteria (Additional file 2, Table S2). Studies deemed potentially eligible underwent full-text review by the same reviewers. Any disagreements were resolved through discussion; if consensus could not be reached, a third reviewer served as an arbitrator. The study selection process is summarized in Figure 1.

### Data extraction

Data were extracted independently by two reviewers (AM and BN) using a standardized form developed in Microsoft Excel. Extracted data included study characteristics (author, year, country, setting, study design, and sample size), population details (type of mobile population, age, sex, clinical status), diagnostic methods used (microscopy, rapid diagnostic tests, PCR), mutations mediating antimalarial resistance detected in PfK13, PfCRT, PfMDR1, PfDHFR, and PfDHPS, and relevant risk factors such as treatment access, diagnostic availability, health-seeking behavior, and nature of mobility. Any discrepancies in data extraction were resolved through consensus or consideration of a third reviewer.

### Risk of bias assessment

Risk of bias was assessed independently by two reviewers using the Joanna Briggs Institute Critical Appraisal Checklist [35]. Key domains included sampling methods, representativeness of the population, reliability of diagnostic techniques, completeness of outcome data, and transparency of reporting. Each study was classified as having low, moderate, or high risk of bias. The overall strength of the body of evidence was evaluated using the GRADE approach [36].

### Effect measures

The primary effect measure was the proportion of samples harbouring single nucleotide polymorphisms mediating antimalarial drug resistance, including non-validated candidate mutations, reported with 95% confidence intervals. Secondary outcomes were the prevalence of *P. falciparum* infection and associations between mobility-related risk factors and infection or resistance, expressed as odds ratios, relative risks, or descriptive summaries.

### Data synthesis and analysis

Both narrative and quantitative syntheses were conducted. Due to inadequate and non-standardized data on mobile populations, we pooled studies across time periods and regions. This approach enabled us to identify overall patterns rather than location or time-specific prevalence estimates. The narrative syntheses summarized the prevalence of *P. falciparum* infection, the distribution of resistance markers across the global regions as defined by WHO, and key risk factors. Quantitative pooling was undertaken to describe aggregate patterns across heterogeneous epidemiological contexts, recognizing that the included studies spanned different time periods and transmission settings. A meta-analysis was performed using the metaprop command in Stata version 17.0 (StataCorp, College Station, TX, USA) to calculate the pooled prevalence of *P. falciparum* infection and the prevalence of drug-resistance mutations. Subgroup analyses were conducted according to geographic region and type of mobile population. Heterogeneity was assessed using the I^2^ statistic, with values of <50%, 50–75%, and >75% indicating low, moderate, and high heterogeneity, respectively [37]. Data on molecular markers mediating antimalarial resistance were synthesized descriptively due to heterogeneity in reporting across studies, including differences in genes assessed, mutation definitions, and outcome measures. Mutation classifications were harmonized according to the WHO compendium of molecular markers for antimalarial drug resistance to ensure consistent categorization across studies [38].

### Missing data

For variables not reported in the original studies, “NR” (not reported) was recorded in the extraction form. Authors of primary studies were contacted to obtain missing data, but none responded.

### Publication bias

Potential publication bias was evaluated through visual inspection of funnel plots, with particular attention to asymmetry and missing studies at the lower end of the distribution, which may indicate underreporting of small or non-significant studies [39]. In addition, Egger’s regression test was conducted to statistically assess funnel plot asymmetry [40].

### Ethical approval

All data analyzed in this review were obtained from previously published and publicly available studies; therefore, ethical approval and informed consent were not required.

## Results

A total of 320 records were identified through database searching. After removal of 126 duplicates, 194 records were screened by title and abstract. An additional three records were identified through citation searching. Following screening, 145 full-text articles were assessed for eligibility, of which 26 met the inclusion criteria and were included in the final synthesis (Fig. 1). The substantial reduction from initial retrieval to final inclusion reflects the specificity of the eligibility criteria and the limited number of studies reporting on mobile populations in the context of *P. falciparum drug resistance*.

### Characteristics of studies reporting *P. falciparum* prevalence among mobile populations in malaria-endemic countries

The 26 included studies were conducted across 10 countries: China, Cambodia, Myanmar, Vietnam, Thailand, Qatar, Uganda, Zambia, Mali, and Suriname. Due to limited and non-standardized data, studies were pooled across time periods and regions, allowing characterization of overall patterns rather than precise location- or time-specific prevalence estimates. While this approach provides broad epidemiological insights, it may obscure important temporal and geographic variation and should be considered when interpreting the findings.

Sixteen studies were conducted in the Western Pacific region; three each in the African, South-East Asia, and Eastern Mediterranean regions; and one in the Region of the Americas. Collectively, these studies included 7,217 participants representing diverse mobile populations, including refugees, migrant workers, travellers, forest goers, gold miners, and nomadic groups. Most studies (25/26; 96.2%) employed cross-sectional designs and primarily enrolled symptomatic individuals. The predominance of symptomatic and cross-sectional studies may bias prevalence estimates and limit inference regarding asymptomatic infections and transmission dynamics.

The highest prevalence of *P. falciparum* infection was observed in the African Region: 44.9% among asymptomatic refugees in Uganda [27], 33.9% among symptomatic refugees in Zambia [41], and 27.0% among symptomatic nomadic populations in Mali [42]. In the Eastern Mediterranean Region, studies in Qatar reported a prevalence of 20.2% among symptomatic migrant workers from Africa and the Indian subcontinent [43–45]. In the Western Pacific Region, prevalence among symptomatic travellers and migrant workers ranged from 8.8% [46] to 16.1% [47]. These differences likely reflect variations in transmission intensity, diagnostic approaches, and population characteristics across regions. In South-East Asia, prevalence data for Myanmar gold miners were not reported [48], and in the region of the Americas, prevalence estimates for symptomatic gold miners in Suriname were unavailable [49] (Table 3).

### *P. falciparum* pooled prevalence and subgroup analysis

**Figure 2:**
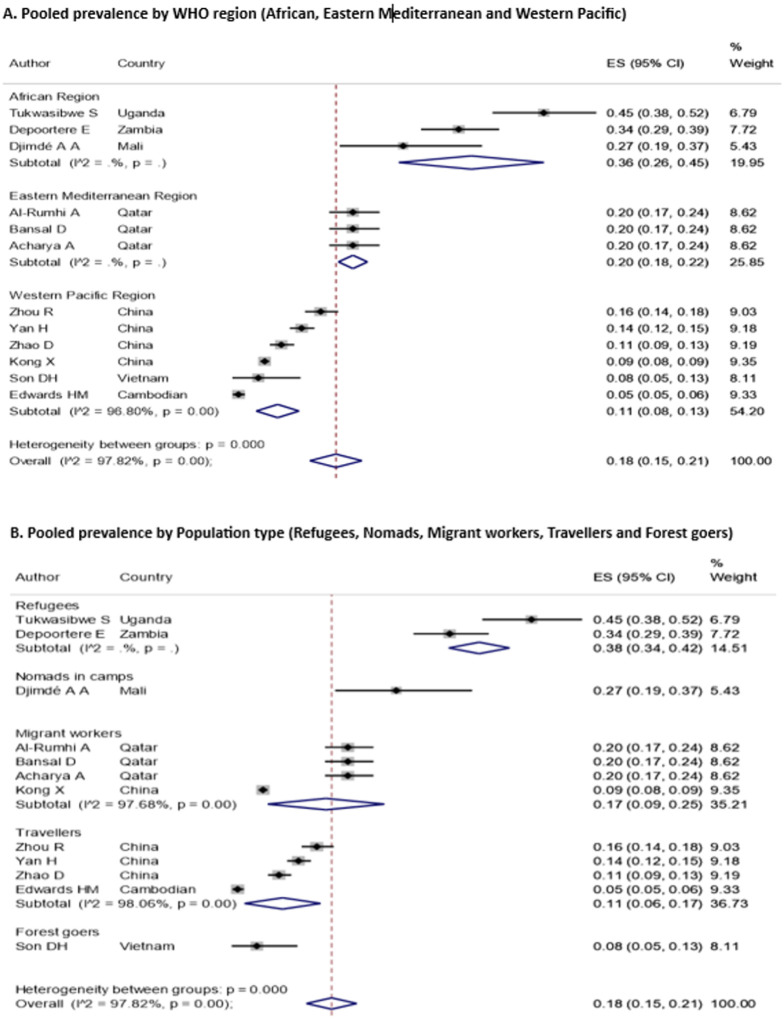
Forest plots representing stratified Pooled Prevalence of P. falciparum among Mobile populations in malaria-endemic countries. **A:**Stratified by WHO geographic region. **B:** Stratified by mobile population type. Each gray square with a central black dot represents the estimated prevalence of P. falciparum from an individual study. Horizontal lines represent 95% confidence intervals. The diamond symbol denotes the pooled prevalence estimate. The vertical line represents the pooled prevalence estimate across studies. Studies to the left indicate lower prevalence, while those to the right indicate higher prevalence than the pooled estimate.

A meta-analysis of 12 studies with extractable prevalence data yielded an overall pooled prevalence of 18% (95% CI: 15–21%) among mobile populations (Fig. 2). The forest plot demonstrates substantial variability in prevalence estimates across individual studies, as reflected by the wide confidence intervals and dispersion of point estimates. While this pooled estimate indicates a considerable burden of infection, it should be interpreted in the context of heterogeneity in study designs, diagnostic methods, and population characteristics.

Subgroup analyses revealed significant heterogeneity across WHO regions and population types. The overall heterogeneity was high (I^2^ = 97.8%, p < 0.001), indicating substantial between-study variability and supporting the use of a random-effects model. This high degree of heterogeneity suggests important underlying epidemiological and methodological differences between studies. By region, the African region had the highest pooled prevalence (36%, 95% CI: 26–45%), followed by the Eastern Mediterranean (20%, 95% CI: 18–22%) and Western Pacific (11%, 95% CI: 8–13%) regions. As shown in Figure 2A, studies from the African region consistently report higher prevalence estimates with wider confidence intervals, suggesting both higher transmission intensity and greater variability. These regional differences likely reflect variation in malaria transmission intensity, access to healthcare, and diagnostic practices. By population type, refugees had the highest pooled prevalence (38%, 95% CI: 34–42%), followed by nomadic individuals (27%, 95% CI: 19–37%), migrant workers (17%, 95% CI: 9–25%) and travellers (8%, 95% CI: 5–13%). The forest plot indicates that studies involving refugees cluster around higher prevalence estimates with relatively narrower confidence intervals, suggesting more consistent findings across these populations. In contrast, studies of travellers and migrant workers show lower prevalence estimates but greater dispersion, reflecting variability in exposure risk and study contexts.

Study weighting indicates that larger studies, particularly from the Western Pacific region, contributed substantially to the overall pooled estimate and may have influenced it toward lower prevalence values. The higher prevalence observed among refugees and nomadic populations may be attributable to increased exposure to malaria vectors, limited access to preventive interventions, and barriers to timely diagnosis and treatment.

### Prevalence and distribution of *P. falciparum* drug resistance mutations in mobile populations across malaria-endemic regions

The prevalence of *P. falciparum* drug-resistance mutations among mobile populations varied across WHO malaria-endemic regions. We studied sequences of five proteins with known mutations associated with resistance, PfK13, PfCRT, PfMDR1, PfDHFR, and PfDHPS. For synthesis, mutation data were extracted as reported in the original studies and subsequently classified by the review team using evidence-based criteria aligned with the WHO compendium of molecular markers for antimalarial drug resistance [38]. These mutations are key molecular determinants of resistance to artemisinins, ACT partner drugs, aminoquinolines and antifolate therapies, forming the backbone of current malaria surveillance frameworks. Because studies differed substantially in sampling year, geographic origin of infections, and mutations screened, results were synthesized to describe overall mutation patterns rather than precise temporal or geographic prevalence. This approach identifies broad resistance trends but limits inference on the evolution and spread of specific mutations over time and across regions.

Mutations mediating antimalarial resistance in all five resistance-associated proteins were widely reported across mobile populations (Table 4). For PfK13, mutations were further grouped into validated, candidate, and potential categories, following WHO criteria, whereas mutations in PfCRT, PfMDR1, PfDHFR, and PfDHPS were classified as known resistance markers or other reported variants. The distribution of mutations indicates both historical resistance (e.g., PfCRT, PfDHFR, PfDHPS) and emerging or evolving resistance (e.g., PfK13). Validated and candidate PfK13 mutations associated with partial artemisinin resistance, as defined by WHO evidence frameworks, including F446I, C469Y, R561H, C580Y, and A675V were reported in Africa and Asia, with no PfK13 mutations reported in the included South American study (Suriname). The PfCRT K76T mutation was widely detected across regions, indicating sustained chloroquine resistance despite drug withdrawal in many regions. This persistence likely reflects continued drug pressure, cross-resistance, or minimal fitness cost. PfMDR1 mutations, including N86Y, Y184F, and D1246Y, which modulate susceptibility to ACT partner drugs such as amodiaquine, lumefantrine and mefloquine, were frequently observed, highlighting potential selection pressure from ACT use among mobile and source populations.

Frequent detection of antifolate resistance mutations in PfDHFR (C59R, N51I, S108N, I164L) and PfDHPS (A437G, K540E, A581G) was observed across multiple regions, with pooled estimates indicating high prevalence (Table 5). The frequent detection of combined PfDHFR–PfDHPS mutations suggests persistence of sulfadoxine–pyrimethamine resistance, with implications for intermittent preventive treatment strategies that rely on sulfadoxinepyrimethamine. Several studies reported multiple mutations within single isolates, indicating potential multidrug resistance profiles. However, variation in molecular methods, mutation panels, and reporting formats limited direct comparability. Additionally, the lack of linked clinical outcome data precluded assessment of genotype–phenotype relationships and the clinical relevance of detected mutations.

### Pooled prevalence and distribution of *P. falciparum* drug resistance mutations among mobile populations in malaria-endemic regions

The pooled prevalence and distribution of drug resistance–associated mutations in *P. falciparum* varied markedly across regions and mobile population groups (Tables 4 and 5; Additional file 3), reflecting differences in antimalarial drug selection pressure and patterns of population mobility. These findings underscore the complex interplay between drug pressure, transmission intensity, and human movement in shaping resistance patterns across regions.

Regionally, mutations in the artemisinin resistance marker PfK13 exhibited substantial heterogeneity. The WHO Western Pacific region showed the most frequent reporting of validated PfK13 mutations, driven by consistent detection of variants such as C580Y, and R539T, in mobile populations in Cambodia, and Vietnam [63, 64]. In some settings, high prevalence of C580Y was observed among forest-goers in Vietnam, indicating established artemisinin partial resistance [63]. This pattern aligns with the Greater Mekong Sub-region as a historical epicenter of artemisinin resistance emergence and spread.

In the Western Pacific region, PfK13 mutations were frequently identified among imported infections in returning migrant workers and travelers in China, though generally at low frequencies and comprising a mixture of validated and non-validated variants [46, 50, 51, 55, 60], reflecting diverse geographic sources of infection. The presence of mixed variants highlights ongoing diversification and potential introduction of novel alleles through human movement.

In the African Region, PfK13 mutations associated with partial artemisinin resistance were less prevalent but notable. Variants including C469Y and A675V in Uganda and R561H and C469F in imported African infections were reported among refugees, travelers, and migrant workers [27, 55, 60], indicating early signals of artemisinin partial resistance and underscoring the need for strengthened genomic surveillance. In contrast, studies from the Eastern Mediterranean Region, primarily involving imported infections among migrant workers in Qatar, showed limited detection of validated PfK13 mutations, with most variants of uncertain functional significance [44, 45]. This likely reflects the imported nature of infections and heterogeneous source populations.

The prevalence of the chloroquine resistance marker PfCRT K76T varied widely across studies, largely reflecting the time periods during which data were collected. Earlier studies conducted during or shortly after widespread chloroquine use such as among gold miners in Suriname [49] and nomadic populations in Mali [42], reported high prevalence. In contrast, more recent studies, particularly from Africa, have documented substantial declines in K76T prevalence following chloroquine withdrawal, although moderate prevalence persists in some migrant populations and imported infections in Asia [42, 50, 53]. These findings suggest that while chloroquine-resistant parasites were historically widespread, their prevalence has declined in many endemic African settings, with continued detection likely reflecting residual transmission, importation, or localized selection pressures.

Similarly, PfMDR1 mutations were frequently reported across regions; however, their distribution is also strongly influenced by temporal trends. The N86Y mutation, once highly prevalent in Africa during the chloroquine era, has declined considerably in recent years, consistent with changing antimalarial treatment policies. Nevertheless, N86Y and Y184F were commonly reported in earlier studies and in infections of African origin, often co-occurring, with D1246Y observed at lower frequencies [27, 49, 53]. These mutations remain relevant due to their potential influence on partner drug efficacy, particularly lumefantrine susceptibility. Additional or less-characterized PfMDR1 variants were typically detected at low frequencies and are of uncertain clinical significance [45, 53].

Antifolate resistance markers in PfDHFR and PfDHPS were highly prevalent. The PfDHFR triple mutant haplotype (N51I, C59R, S108N) was consistently reported across studies in both African and Western Pacific populations [27, 52, 56], while I164L remained relatively uncommon. PfDHPS mutations, particularly A437G and K540E, were widely distributed [44, 53], with higher-order resistance haplotypes (A581G, A613S) frequently reported among imported infections in China and Qatar [44, 53]. These findings reflect sustained antifolate drug pressure and limited reversion following policy changes.

Across mobile population groups, as described in individual studies, distinct resistance profiles were observed. Refugees exhibited high prevalence of PfMDR1 mutations (N86Y, Y184F) and near fixation of the PfDHFR triple mutant [27, 41], while PfK13 mutations were moderate (C469Y, A675V) [27]. International travellers showed higher PfK13 prevalence, reflecting exposure to artemisinin-resistant parasites in Southeast Asia, alongside heterogeneous PfCRT and PfMDR1 profiles [53, 54, 57, 63, 64]. Nomads had high PfCRT K76T prevalence and moderate PfDHFR prevalence [42]. Migrant workers displayed intermediate PfMDR1 prevalence (N86Y, Y184F) and common PfCRT K76T prevalence, with low-frequency of PfK13 mutations (R561H, C469F) [45, 50, 51, 55, 56, 60], and high PfDHFR–PfDHPS mutation prevalence [52, 56], suggesting these populations may facilitate regional maintenance and spread of resistance markers.

Certain populations, including forest-goers and gold miners, had insufficient data for pooled estimates. However, individual studies revealed localized resistance hotspots: gold miners in Suriname showed accumulation of multiple *PfMDR1* and *PfDHFR* mutations alongside persistent *PfCRT* K76T [49], while forest-goers in Vietnam exhibited high prevalence of *PfK13* C580Y [63]. These localized hotspots may represent focal points for the emergence and onward spread of resistance.

Overall, antifolate resistance markers (PfDHFR, PfDHPS) were highly prevalent across mobile populations, whereas PfK13, PfCRT, and PfMDR1 mutations showed heterogeneous distribution, reflecting differential selection pressures and changes in prevalences over time. Given study heterogeneity in design, sampling, geographic origin, and mutation assessment, pooled estimates should be interpreted as indicative of broad epidemiological trends rather than precise prevalence. Future studies integrating genomic, clinical, and epidemiological data are needed to refine genotype–phenotype relationships and guide targeted interventions.

### Factors associated with *P. falciparum* infection and resistance among mobile populations in malaria endemic settings.

Only five (18.5%) of the included studies investigated factors associated with *P. falciparum* infection among mobile populations, highlighting a significant evidence gap in understanding the drivers of infection risk in these groups. Along the Cambodian border, Edwards et al. [64] reported higher infection risk among men, individuals engaged in manual labor (particularly forest-related work), those crossing borders during August–September, individuals with less frequent crossings (fewer than once per week), and forest-goers. These factors are consistent with increased exposure to vector habitats and occupational risk. Across other studies, descriptive findings indicated that forest-goers, miners, refugees, and travelers had high infection burdens, although statistical associations were not consistently examined. The lack of standardized analytical approaches limits comparability and the strength of inference across studies.

Few studies examined factors associated with molecular resistance markers. In Qatar, Acharya et al. [43] found no significant differences in PfCRT or PfMDR1 genotypes by age, gender, hemoglobin level, or parasitemia among imported cases. Similarly, studies among returning migrant workers in Henan Province, China, reported no significant variation in the prevalence of PfK13, PfMDR1, PfCRT, PfDHFR, or PfDHPS mutations by demographic characteristics or sampling period [47, 59, 62]. These findings suggest that host demographic factors may play a limited role in shaping resistance profiles compared to parasite origin and drug selection pressure.

A critical overarching pattern emerging from these studies is that the source location of infection is substantially more influential than the location where the individual is tested or diagnosed. Nearly all malaria cases detected among returnees in China and imported cases in Qatar originated from African countries, where specific resistance patterns are well established. For example, antifolate resistance markers in PfDHFR and PfDHPS were almost fixed in many African regions [52, 56, 58]; chloroquine-resistance markers such as PfCRT K76T were prevalent in some areas [50, 53]; and PfMDR1 mutations associated with ACT partner-drug selection most commonly N86Y, Y184F, and D1246Y were widespread [27, 49, 53]. These patterns reflect sustained drug pressure and established resistance landscapes in source regions. Consequently, the uniformity of resistance profiles observed in destination countries such as China [46, 50, 51, 55, 60] and Qatar [43–45] reflects the molecular epidemiology of parasites circulating in migrants’ countries of origin, rather than local transmission dynamics.

### Heterogeneity across studies

Substantial statistical heterogeneity was observed across the included studies. The overall I^2^ was 97.8% (p < 0.001), indicating considerable variability in *P. falciparum* prevalence estimates. This high level of heterogeneity suggests that pooled estimates are influenced by significant between-study differences rather than random variation alone. High heterogeneity persisted in stratified analyses, with an I^2^ of 95.6% in the Western Pacific Region, 97.7% among studies of migrant workers (p < 0.001), and 98.1% among studies of international travelers (p < 0.001) (Additional file 4, Table S4). The consistency of high I^2^ values across subgroups indicates that heterogeneity is not confined to specific regions or populations but is a universal feature of the dataset. This heterogeneity likely reflects differences in study populations, geographic settings, transmission intensity, time periods, and diagnostic methods. Additional contributors may include variation in study design, sampling strategies, and definitions of mobile populations across studies. Therefore, pooled estimates should be interpreted as contextual summaries rather than uniform measures, and findings support cautious interpretation of meta-analytic results. Standardized study designs and reporting are needed in future research to improve comparability.

### Risk of bias in the included studies

Methodological quality and reporting bias were assessed as high in 19.2% (5/26) of studies, indicating that a notable proportion may have limitations affecting the reliability of their findings. Potential selection bias was identified in 11.5% (3/26) of studies, where genotyping success rates were below 90% (Additional file 5, Table S5). Low genotyping success may underrepresent certain parasite populations and introduce bias in estimates of resistance marker prevalence.

### Publication bias assessment

Visual inspection of the funnel plot based on *P. falciparum* prevalence estimates revealed asymmetry around the central line, suggesting possible publication bias. Such asymmetry may indicate selective reporting of studies with higher or statistically significant prevalence estimates. This was further supported by Egger’s regression test, which yielded a coefficient of 6.68, z = 3.64, and P = 0.0003 (Additional file 6, Figure S6). The statistically significant result from Egger’s test provides quantitative evidence of small-study effects or publication bias, although these findings should be interpreted cautiously given the high heterogeneity across studies.

## Discussion

This systematic review and meta-analysis synthesized evidence on *P. falciparum* infection prevalence and molecular markers of antimalarial drug resistance, including WHO-validated mutations and other candidate markers, as well as reported variants, among mobile populations in malaria-endemic regions. The findings suggest an important role for human mobility, including labor migration, forced displacement, nomadism, and cross-border travel in sustaining malaria transmission and facilitating the geographic spread of drug-resistant parasites. These results reinforce the concept of mobile populations as epidemiological connectors linking transmission settings with differing intensity and resistance profiles. Although global malaria incidence has declined in several regions, mobile populations continue to function as epidemiological bridges between high- and low-transmission settings, complicating elimination efforts and enabling parasite dispersal across borders. These dynamics challenge traditional geographically bounded control strategies and highlight the need for mobility-informed approaches to malaria surveillance and control.

The pooled prevalence of *P. falciparum* infection among mobile populations was 18% (95% CI: 15–21%), higher than that reported in general migrant populations (8.3%) [66] but lower than estimates among displaced populations in sub-Saharan Africa (34.9%) [28]. This burden likely reflects a convergence of structural vulnerability, occupational exposure, and limited access to timely diagnosis and treatment, illustrating how social and health system inequities intersect with biological risk to sustain transmission. Regional variation closely mirrored underlying transmission intensity. The African region exhibited the highest prevalence (~36%), consistent with entrenched transmission, ongoing antimalarial drug resistance, and widespread insecticide resistance [67]. The lowest prevalence was observed in the Greater Mekong subregion, a cross-border malaria elimination zone spanning parts of the WHO South-East Asia and Western Pacific regions [68]. Intermediate prevalence in the Western Pacific (~11–12%) and Eastern Mediterranean (~20%) regions likely reflects a combination of imported infections and residual transmission along major mobility corridors, further supporting the role of mobility in redistributing infection risk across regions.

Marked differences were observed across population groups. Refugees consistently experienced the highest prevalence (38%), likely driven by displacement-related disruption of health systems, overcrowded living conditions, and limited access to preventive interventions [69–71]. Although migrant workers and international travelers exhibited comparatively lower prevalence, their high mobility positions them as important conduits for introducing *P. falciparum* into malaria-free or low-endemic settings, thereby sustaining transmission and reintroduction risk [72]. Thus, even groups with lower prevalence may exert disproportionate epidemiological impact due to their movement patterns.

The distribution of molecular markers associated with antimalarial drug resistance, including validated markers and other reported variants, closely paralleled these epidemiological patterns, reinforcing the vulnerability of mobile populations and their role in disseminating drug-resistant parasites. Validated *PfK13* mutations, including C580Y, R561H, and R539T, were most prevalent in South-East Asia, particularly among forest-goers and cross-border populations [63, 64], consistent with established foci of artemisinin resistance. The detection of validated *PfK13* mutations such as C469Y and A675V in Uganda [27] and R561H in other African-origin infections [55, 60] indicates the presence of artemisinin resistance–associated alleles outside Southeast Asia, raising concern about potential regional spread and underscoring the need for strengthened genomic surveillance. In contrast, non-validated or low-evidence *PfK13* mutations among migrants returning to China and Gulf countries [73] reflect importation of diverse parasite lineages through human movement rather than evidence of local emergence or diversification of resistance.

High prevalence of validated PfCRTmutations associated with chloroquine resistance was observed across multiple regions, particularly among African refugees, migrant workers, and imported infections [27, 45, 61]. Despite the withdrawal of chloroquine as first-line therapy in most endemic settings, persistence of PfCRT K76T indicates continued circulation of resistant parasites. However, much of the evidence for high prevalence derives from studies conducted more than a decade ago or during the early post-chloroquine withdrawal period, whereas more recent data from Africa demonstrate substantial declines in prevalence following drug policy change[74]. Similarly, *PfMDR1* mutations, particularly N86Y and Y184F, were detected at consistently high frequencies across multiple populations, mostly from studies conducted some years ago [27, 45, 61]. These mutations modulate susceptibility to ACT partner drugs, and their widespread occurrence suggests ongoing selection pressure with potential implications for treatment efficacy. Additional *PfMDR1* variants (including D1246Y and other less frequent mutations listed in Table 4) were generally rare and represent minor or background polymorphisms rather than dominant resistance haplotypes across populations.

Antifolate resistance markers were highly prevalent across nearly all mobile populations. *PfDHFR* mutations (N51I, C59R, S108N) were consistently reported at very high frequency, and pooled estimates indicate very high prevalence across multiple populations, while *PfDHPS* mutations (A437G, K540E) commonly co-occurred [27]. The persistence of these mutations has important implications for sulfadoxine–pyrimethamine–based interventions, including intermittent preventive treatment. Refugees exhibited high prevalence of antifolate resistance markers, while estimates in nomadic populations were lower and based on limited data [27, 75]. Migrant workers and travellers, despite lower infection prevalence, remain important vectors for cross-border dissemination of resistant haplotypes [45]. Across these loci, reported mutations include both validated resistance markers and additional variants, some of which represent polymorphisms or haplotype components rather than independently validated resistance determinants.

Behavioral and occupational factors further amplify infection risk. Male sex, engagement in manual labor, particularly forest-related activities, cross-border movement during peak transmission periods, and prolonged stays in endemic areas were consistently associated with higher infection prevalence [64]. These findings highlight the importance of targeting high-risk occupations and behaviors in intervention strategies. The substantial heterogeneity observed across studies reflects not only methodological differences but also the inherently dynamic nature of malaria transmission in mobile populations. Differences in study settings, participant characteristics, sampling periods, mutation panels, and infection origins indicate that pooled estimates represent broad epidemiological patterns rather than precise measures. Importantly, this heterogeneity reflects the complexity of malaria epidemiology in mobile populations rather than merely a methodological limitation.

### Implications of study findings for malaria control and elimination

These findings indicate that mobile populations likely act as reservoirs of *P. falciparum* drug-resistant parasites, facilitating both local transmission and cross-border dissemination. This underscores the need to explicitly integrate human mobility into malaria elimination frameworks, particularly in regions with porous borders and high population movement.

Effective control strategies should prioritize strengthening border health systems, including targeted screening and case management at points of entry, alongside deployment of mobile diagnostic and treatment services to reach hard-to-access populations. Real-time genomic surveillance integrated with routine epidemiological monitoring would enhance early detection of resistance hotspots and emerging transmission clusters. In addition, improved cross-border collaboration and data sharing between endemic and non-endemic countries will be critical for coordinated response and timely containment of imported infections.

Expanding access to prevention and treatment services for mobile populations remains essential to reduce both infection risk and antimalarial drug selection pressure. Tailored interventions, including mobile vector control strategies and periodic targeted mass screening in high-risk groups such as refugees, migrant workers, and forest-related occupational groups, may further reduce transmission. Integrating molecular surveillance with mobility and travel-history data offers a particularly powerful approach for adaptive and responsive malaria control strategies.

While interventions focus on malaria transmission and drug resistance, the evidence suggests wider health consequences. Although resistance was once considered a low-transmission settings issue, its emergence in high-burden areas like northern Uganda raises concerns for BL. In settings where malaria and BL overlap, artemisinin resistance [10, 15, 76] may cause treatment failure or incomplete parasite clearance, leading to the prolonged immune stimulation hypothesized to influence BL risk. Continued circulation of resistant parasites could undermine malaria control and reverse the recent decline of BL incidence in sub-Saharan Africa [77]. While this review does not directly assess this link, it identified a vital area for interdisciplinary research into the health impacts of persistent malaria in mobile populations.

### Study Limitations and Strengths

Interpretation of these findings should consider several limitations. Most included studies were cross-sectional and facility-based, limiting causal inference and potentially underestimating infection burden in hard-to-reach populations. The prevalence of some drug resistance markers has changed substantially over the 25 years covered, and temporal variation likely contributed to differences between studies. Heterogeneity in diagnostic approaches and molecular methods may also have influenced estimates. The aggregation of studies across diverse geographic regions and time periods introduced substantial heterogeneity (I^2^ = 97.8%, p < 0.001), which may obscure fine-scale patterns. Evidence of small-study effects further necessitates cautious interpretation of pooled estimates. These limitations highlight the need for standardized methodologies in future research.

Despite these limitations, this study has several notable strengths. It represents the first comprehensive synthesis focusing specifically on mobile populations, integrating both epidemiological and molecular data across regions. The inclusion of diverse population groups enhances the generalizability of findings, while the identification of behavioral and occupational risk factors provides actionable insights for targeted interventions.

## Conclusion

Mobile populations are likely to contribute to malaria transmission dynamics and the distribution of drug resistance, although the extent of their impact cannot be determined from the available evidence. The observed prevalence of *P. falciparum* infection and drug resistance markers among refugees, nomadic groups, and migrant workers highlights their potential epidemiological relevance. These populations may therefore warrant consideration in surveillance and intervention strategies, particularly in settings with ongoing transmission or cross-border movement. Strengthening mobility-informed surveillance, improving cross-border collaboration, and ensuring equitable access to prevention and treatment services could support malaria control efforts. Further longitudinal and population-based studies are needed to better quantify the role of mobile populations and to inform targeted interventions for sustainable progress toward malaria elimination.

## Supplementary Material

Supplementary Files

This is a list of supplementary files associated with this preprint. Click to download.

- Additionalfile1.docx
- Additionalfile2.xls
- Additionalfile3.docx
- Additionalfile4.docx
- Additionalfile5.docx
- Additionalfile6.docx

## Figures and Tables

**Table 1.** The PECOST framework

Population	Mobile populations in malaria-endemic countries
Exposure	*P falciparum* infection
Comparator	None
Outcomes	**Primary:** Resistance markers; **Secondary:** Infection prevalence, and risk factors
Settings	Malaria-endemic regions
Time	January 2000 to October 2025

**Table 2.** Definition of mobile populations

Population type	Definition / Description
**Refugees**	Individuals who have fled their home country due to conflict, persecution, or disaster and reside temporarily in host countries or camps.
**Migrant workers**	People who relocate temporarily or seasonally from one region or country to another for employment.
**Travellers**	Individuals moving temporarily between low and high-malaria transmission areas for leisure, business, or other purposes.
**Nomadic populations**	Communities moving regularly within a region for livelihood, such as pastoralists.
**Gold miners**	Individuals temporarily migrating to work in gold mining sites
**Forest goers**	Individuals frequently entering forested areas for livelihood activities (hunting, gathering, or logging).

**Table 3.** Characteristics of studies which reported *P. falciparum* prevalence among mobile populations in malaria-endemic countries

First Author	Year	Country	Region	Study Design	Mobile Population	Sample Size	Clinical Status	*P. falciparum* prevalence (%)
Tukwasibwe S [27]	2024	Uganda	African Region	Cross-sectional	Refugees from DRC and South Sudan in Uganda	421	Asymptomatic	44.9
Wang X [50]	2022	China	Western Pacific	Cross-sectional	Migrants from Nigeria to China	165	Symptomatic	NR
Kong X [46]	2022	China	Western Pacific	Cross-sectional	Migrant returnees from Africa and South East Asia	425	Symptomatic	8.8
Cheng W [51]	2021	China	Western Pacific	Cross-sectional	Migrant workers returning from Africa	106	Symptomatic	NA
Yan H [52]	2021	China	Western Pacific	Cross-sectional	Chinese migrants from Africa	159	Symptomatic	NR
Zhao D [53]	2021	China	Western Pacific	Cross-sectional	Chinese travellers returning from Nigeria	167	Symptomatic	10.9
Yan H [54]	2020	China	Western Pacific	Cross-sectional	Travellers returning from Africa	153	Symptomatic	13.6
Wang X [55]	2020	China	Western Pacific	Cross-sectional	Migrant workers from Africa, SEA and Oceania	485	Symptomatic	NR
Jiang T [56]	2020	China	Western Pacific	Cross-sectional	Migrant workers returning from Africa and SEA	303	Symptomatic	NR
Al-Rumhi A [45]	2020	Qatar	Eastern Mediterranean	Cross-sectional	Migrant workers from Indian subcontinent and Africa	118	Symptomatic	20.2
He Y [57]	2019	China	Western Pacific	Cross-sectional	Travellers across China-Myanmar border	72	Symptomatic	NR
Bansal D [44]	2019	Qatar	Eastern Mediterranean	Cross-sectional	Migrants from East, West and Central Africa and India	118	Symptomatic	20.2
Xu C [58]	2019	China	Western Pacific	Cross-sectional	Migrant workers returning from Africa	254	Symptomatic	NR
Zhou R [47]	2019	China	Western Pacific	Cross-sectional	Travellers returning from Africa (Angola)	213	Symptomatic	16.1
Yang C [59]	2019	China	Western Pacific	Cross-sectional	Migrant workers from Africa	632	Symptomatic	NR
Zhang T [60]	2018	China	Western Pacific	Cross-sectional	Chinese migrant workers who returned from Africa to Anhui, China	460	Symptomatic	NR
Yao Y [61]	2018	China	Western Pacific	Cross-sectional	Chinese migrant workers returning from Africa and South East Asia to Wuhan, China	230	Symptomatic	NR
Acharya A [43]	2018	Qatar	Eastern Mediterranean	Cross-sectional	Migrant workers from East, West and Central Africa and India	118	Symptomatic	20.2
Yang C [62]	2017	China	Western Pacific	Cross-sectional	Migrant workers who returned from Africa to Henan Province	483	Symptomatic	NR
Son DH [63]	2017	Vietnam	Western Pacific	Cross-sectional	Forest goers	150	Asymptomatic	8
Nyunt MH [48]	2017	Myanmar	South-East Asia	Cross-sectional	Migrant gold mine workers in Myanmar	100	Symptomatic	NR
Edwards HM [64]	2015	Cambodia	Western Pacific	Cross-sectional	Travellers across Cambodian borders	174	Mixed	5.4
Adhin MR [49]	2014	Suriname	Region of the Americas	Cross-sectional	Gold miners	28	Symptomatic	NR
Feng J [65]	2014	China	Western Pacific	Cross-sectional	Migrant workers returned from Ghana to, China	118	Symptomatic	NR
Depoortere E [41]	2005	Zambia	African region	RCT	Refugees from Angola, DRC, Rwanda and Burundi in Zambia	169	Symptomatic	33.9
Djimdé AA [42]	2004	Mali	African region	Cross-sectional	Nomadic people in camps	86	Symptomatic	27

**NR** = not reported; prevalence estimates are based on diagnostic methods reported in individual studies. “**Mixed**” indicates inclusion of both symptomatic and asymptomatic participants.

**Table 4.** Distribution of known and other mutations in five *P. falciparum* genes associated with antimalarial drug resistance

First Author (Year)	Country	K13 validated	K13 candidate	K13 potential	PfCRT Known	PfCRT other	PfMDR1 Known	PfMDR1 other	PfDHFR Known	PfDHFR other	PfDHPS Known	PfDHPS other
Tukwasibwe S (2024) [27]	Uganda	C469Y, A675V	NR	NR	K76T	NR	N86Y	Y184F, D1246Y	N51I, C59R, S108N	I164L	A581G, A437G, K540E	
Wang X (2022) [50]	China	NR	C469F	A676S, P441S, N458D, V650F	K76T	N75K, N75E	N86Y	Y184F	N51I, C59R, S108N	D139N	A581G, S436A, A437G, K540E, A613S,	I431V
Kong X (2022) [46]	China	C580Y	NR	A578S, F439S, F442L, P443A, C473S, S588C, Q613H, S626R, G665S	K76T	M74I	NR	NR	NR	NR	NR	NR
Cheng W (2021) [51]	China	NR	NR	NR	K76T	M74I, N75E	N86Y	Y184F	NR	NR	NR	NR
Yan H (2021) [52]	China	NR	NR	NR	NR	NR	NR	NR	N51I, C59R, S108N	NR	A613S, A581G, S436A, A437G, K540E	I431V
Zhao D (2021) [53]	China	NR	NR	S693F, Q613H	K76T	M74I, N75E	N86Y	Y184F, D1246Y, T1192L	N51I, C59R, S108N	I164L	A613S,A581G, S436A, A437G	I431V
Yan H (2021) [54]	China	NR	NR	V589A	NR	NR	NR	NR	NR	NR	NR	NR
Wang X (2020) [55]	China	R561H, R622I	NR	A578S, P441S, K503E, D464E, V650F, N694K	K76T	NR	NR	NR	NR	NR	NR	NR
Jiang T (2020) [56]	China	NR	NR	NR	NR	NR	NR	NR	N51I, C59R, S108N	I164L	A613S, A581G, S436A, A437G, K540E	NR
Al-Rumhi A (2020) [45]	Qatar	NR	NR	K108E, H136N, T149S, K189T, N217H, R255K, I354V, E433D, G453A	K76T	C72S	N86Y	Y184F, D1246Y	N51I, C59R, S108N	I164L	A613S, A581G, S436A, A437G, K540E	I43
He Y (2019) [57]	China	F446I, Y493H	NR	A676D, V603E, P574L	K76T	C72S	N86Y	Y184F, S1034I, F938Y, G968A, D90H, V104A, F1226Y	NR	NR	NR	NR
Bansal D (2019) [44]	Qatar	NR	NR	G453A	NR	NR	NR	NR	N51I, C59R, S108N	NR	A613S, A581G, S436A, A437G, K540E	NR
Xu C (2019) [58]	China	NR	NR	NR	NR	NR	NR	I107V	N51I, C59R, S108N	NR	S436A, A437G, K540E, A581G, A613S	I43
Zhou R (2019) [47]	China	NR	NR	M579I, A578S, Q613E	K76T	M74I, N75E	N86Y	Y184F, D1246Y, I107V	I164L, N51I, C59R, S108N	D139Y	A581G S436A, A437G, K540E	NR
Yang C (2019) [59]	China	NR	NR	NR	NR	NR	N86Y	Y184F, D1246Y	NR	NR	NR	NR
Zhang T (2019) [60]	China	NR	C469F	L488V, I683R, L457S, A578S, Q613E, Q613H, P443R, V589I, F434Y, T437N, D462N, I634T, A621S, L619E	K76T	M74I, N75K, N75E	N86Y	NR	NR	NR	NR	NR
Yao Y (2018) [61]	China	R561H	NR	V589I	K76T	C72S, M74I, N75E	N86Y	Y184F	NR	NR	NR	NR
Acharya A (2018) [43]	Qatar	NR	NR	NR	K76T	NR	N86Y	NR	NR	NR	NR	NR
Yang C (2017) [62]	China	R539T, P574L, I543T, Y493H, C580Y	NR	M471I, E556K, L663V, K658Q, F662C, D648Y, A578T, A578S, M579I, V589I, Q613E, K610R	NR	NR	NR	NR	NR	NR	NR	NR
Son DH (2017) [63]	Vietnam	C580Y	NR	NR	NR	NR	NR	NR	NR	NR	NR	NR
Nyunt MH (2017) [48]	Myanmar	C580Y, P574L, M476I	NR	P667T	NR	NR	NR	NR	NR	NR	NR	NR
Edwards HM (2015) [64]	Cambodia	C580Y, R539T	NR	NR	NR	NR	NR	NR	NR	NR	NR	NR
Adhin MR (2014) _[49]	Suriname	NR	NR	NR	K76T	NR	NR	Y184F, S1034C, N1042D, D1246Y	N51I, S108N	NR	NR	NR
Feng J (2014) [65]	China	C580Y, R539T	NR	C580F, R575T, D584V, V692L	K76T	M74I, N75E	N86Y	Y184F, S1034C, D1246Y	NR	NR	NR	NR
Depoortere E (2005) [41]	Zambia	NR	NR	NR	NR	NR	NR	NR	S108N	NR	A437G	NR
Djimdé AA (2004) [42]	Mali	NR	NR	NR	K76T	NR	NR	NR	C59R, S108N	NR	A437G	NR

**NR** = not reported. K13 mutations were classified as validated, candidate, or potential based on harmonisation with the WHO compendium of molecular markers for antimalarial drug resistance. The “K13 potential” category groups non-validated variants with limited or preliminary evidence of association. Mutations in PfCRT, PfMDR1, PfDHFR, and PfDHPS were classified as either known resistance markers or other reported variants according to WHO compendium definitions. “Other” includes polymorphisms and variants with limited or unclear evidence of association with antimalarial drug resistance. Mutations are reported as described in the original studies and may occur as single mutations or within haplotypes associated with resistance phenotypes.

**Table 5.** Pooled prevalence of *P. falciparum* drug resistance mutations among mobile populations in malaria endemic regions

Resistance marker	Migrant workers		Travellers		Refugees		Goldminers		Forest goers		Nomads		All mobile	
	Freq (%)	95% CI	Freq (%)	95% CI	Freq (%)	95% CI	Freq (%)	95% CI	Freq (%)	95% CI	Freq (%)	95% CI	Freq (%)	95% CI
*PfK13*	8	4–11	26	12–40	17	12–24	21	14–30	100**		NR		12	9–15
*PfCRT*	33	24–42	52	16–89	20	14–28	100**		NR		79	67–88	40	29–50
*PfMDRI*	43	26–60	52	34–70	68	59–76	100**		NR		NR		47	34–61
**PfDHFR**	98	96–99	98	97–99	97	92–99	100**		NR		45	27–65	97	96–99
**PfDHPS**	80	68–91	96	94–98	59	50–68	NR		NR		05	01–22	73	61–85

Values marked with ** represent proportions derived from a single study and should be interpreted with caution, as pooled estimates could not be calculated due to limited data and small sample sizes. Due to the lack of multiple data points and small sample sizes, pooled prevalence could not be computed for these subgroups using meta-analysis. NR = not reported; indicates that no data were available for the specified resistance marker within the given population group. Pooled prevalence estimates were generated using random-effects meta-analysis where sufficient data were available; estimates based on single studies are presented descriptively.

## Data Availability

All data generated or analysed during this study are included in this published article and its supplementary information files.

## Abbreviations

ACT: Artemisinin Combination Therapy
AL: Artemether Lumefantrine
GRADE: Grading of Recommendations, Assessment, Development, and Evaluations
BL: Burkitt Lymphoma
IDPs: Internal Displaced Persons
PECOST: Population Exposure Comparator Outcome Settings Time
PCR: Polymerase Chain Reaction
PRISMA: Preferred Reporting Items for Systematic Reviews and Meta-Analyses
RoB: Risk of Bias
SEA: South East Asia
WHO: World Health Organisation
